# Brain MRI and cognitive function seven years after surviving an episode of severe acute malnutrition in a cohort of Malawian children

**DOI:** 10.1017/S1368980018003282

**Published:** 2018-12-03

**Authors:** Natasha Lelijveld, Alhaji A Jalloh, Samuel D Kampondeni, Andrew Seal, Jonathan C Wells, Magdalena Goyheneix, Emmanuel Chimwezi, Macpherson Mallewa, Moffat J Nyirenda, Robert S Heyderman, Marko Kerac

**Affiliations:** 1 Institute for Global Health, University College London, 30 Guildford Street, London WC1N 1EH, UK; 2 Malawi–Liverpool–Wellcome Trust Clinical Research Programme, Blantyre, Malawi; 3 Centre for Global Child Health, Sick Kids Hospital, Toronto, Ontario, Canada; 4 University of Malawi College of Medicine, Blantyre, Malawi; 5 Childhood Nutrition Research Centre, Institute of Child Health, University College London, London, UK; 6 Department of Population Health, London School of Hygiene and Tropical Medicine, London UK; 7 MRC/UVRI Uganda Research Unit, Entebbe, Uganda; 8 Division of Infection & Immunity, University College London, London, UK

**Keywords:** Severe acute malnutrition, Acute malnutrition, Long-term outcomes, Post-discharge, Cognitive function, Brain structure, Malawi

## Abstract

**Objective:**

To assess differences in cognition functions and gross brain structure in children seven years after an episode of severe acute malnutrition (SAM), compared with other Malawian children.

**Design:**

Prospective longitudinal cohort assessing school grade achieved and results of five computer-based (CANTAB) tests, covering three cognitive domains. A subset underwent brain MRI scans which were reviewed using a standardized checklist of gross abnormalities and compared with a reference population of Malawian children.

**Setting:**

Blantyre, Malawi.

**Participants:**

Children discharged from SAM treatment in 2006 and 2007 (*n* 320; median age 9·3 years) were compared with controls: siblings closest in age to the SAM survivors and age/sex-matched community children.

**Results:**

SAM survivors were significantly more likely to be in a lower grade at school than controls (adjusted OR = 0·4; 95 % CI 0·3, 0·6; *P* < 0·0001) and had consistently poorer scores in all CANTAB cognitive tests. Adjusting for HIV and socio-economic status diminished statistically significant differences. There were no significant differences in odds of brain abnormalities and sinusitis between SAM survivors (*n* 49) and reference children (OR = 1·11; 95 % CI 0·61, 2·03; *P* = 0·73).

**Conclusions:**

Despite apparent preservation in gross brain structure, persistent impaired school achievement is likely to be detrimental to individual attainment and economic well-being. Understanding the multifactorial causes of lower school achievement is therefore needed to design interventions for SAM survivors to thrive in adulthood. The cognitive and potential economic implications of SAM need further emphasis to better advocate for SAM prevention and early treatment.

More than 200 million children under 5 years of age worldwide fail to reach their full developmental potential^(^^1^^)^. It has long been recognized that social and environmental factors, including nutrition, have a strong influence on cognitive, language and socio-emotional development^(^^2^^,^^3^^)^. Recent focus on the importance of early-life exposures has resulted in strong global advocacy movements such as Scaling Up Nutrition (SUN), which highlights the long-term impacts of the ‘1st 1000 days of life’^(^^4^^)^. SUN’s main focus is on chronic childhood malnutrition resulting in stunting (low height-for-age): this has well documented adverse consequences for individual, population and societal development^(^^5^^)^. In contrast, the links between acute malnutrition, which is also a major global public health problem, and development have been less well described.

Severe acute malnutrition (SAM) affects at least 17 million children under 5 years of age worldwide^(^^6^^)^. Infants and children in the first 2 years of life are most vulnerable due to a high BMR, increased nutritional requirements due to rapid physical growth and increased risk of infections^(^^7^^)^. Reducing mortality from SAM is still a priority; however, with SAM survival rates increasing, the long-term outcomes also need consideration^(^^8^^)^.

A number of studies have explored potential effects of SAM on brain function and structure, but many of these use old case definitions of SAM, short time scales and diverse, complex testing tools which measure a variety of different outcomes^(^^9^^–^^11^^)^. One pivotal review of studies linking SAM and mental development between 1956 and 1994 concluded that school-age children who suffered from early childhood undernutrition generally had poorer IQ levels, cognitive function, school achievement, and greater behavioural problems than matched controls and, to a lesser extent, siblings^(^^12^^)^. However, no consistent, specific cognitive deficit was found across the studies reviewed.

A more recent review of fifteen studies which included publications from large cohorts in Mauritius and Barbados^(^^13^^–^^15^^)^ found consistent associations between SAM and various cognitive impairments including short-term memory, problem solving, IQ, cognitive processing, working memory and academic skills. However, again there were no studies using current anthropometric definitions of SAM^(^^15^^,^^16^^)^. Moreover, these studies used different and often complex assessment tools which are unsuitable to assess cognitive outcomes in large-scale, field-based, multi-outcome epidemiological studies.

A relatively quick and simple approach is the Cambridge Neuropsychological Testing Automated Battery (CANTAB)^(^^17^^)^, which utilizes touch-screen technology to measure cognitive function in a series of tests. CANTAB tests were recently successfully used in a trial to assess the impact of school feeding on cognitive function in Malawi^(^^18^^)^.

Besides functional changes associated with SAM, there is also interest in possible changes in underlying brain structure. Indeed, early studies using computerized tomography showed that SAM was associated with acute brain changes^(^^19^^)^, some of which resolved after nutritional rehabilitation^(^^20^^)^. Similarly, more recent MRI studies during an episode of SAM showed structural changes including dilated ventricles, cerebral atrophy and periventricular white matter change^(^^21^^,^^22^^)^; some of these features had resolved at 90 d, but it is unknown whether any longer-term changes remain in SAM survivors.

Our study aimed to assess multiple aspects of cognition in the years following treatment for an episode of SAM, including: school achievement; cognitive function as assessed by CANTAB computer-based testing; and brain structure as assessed by MRI scan. As SAM survival increases, this evidence on its long-term outcomes is much needed, not only to shape better short-term interventions but also to better advocate for prevention strategies.

## Methods

This was a longitudinal cohort study which prospectively followed-up survivors of SAM seven years post-discharge from treatment in Blantyre, Malawi, to examine cognitive function and other health outcomes. Sibling and age/sex-matched community controls were recruited for comparison.

### Study setting and participants

Full details of the cohort, as well as additional methods and results on other outcomes, have been described elsewhere^(^^8^^,^^23^^,^^24^^)^. In brief, the cohort originally included all patients admitted to the nutrition ward for treatment of SAM in Queen Elizabeth Central Hospital, Blantyre, Malawi, from 12 July 2006 to 9 March 2007 (1024 children). The median age of the children at admission was 21·5 months (interquartile range 15–32 months). Results of survival and anthropometry at the baseline study and the 1-year follow-up have been described previously^(^^25^^,^^26^^)^. Sibling controls were defined as those closest in age to the SAM survivor (‘case child’), between the ages of 6 and 15·9 years; community controls were defined as a child living in the same community, of the same sex, and within 12 months of age of the case child, randomly selected by spinning a bottle at the case child’s home to select a random direction, then enquiring door-to-door to find the first eligible child. Children who had ever been treated for acute malnutrition were excluded from the control group. Informed written consent was obtained from the child’s parent or guardian; assent was required from the children themselves. One control child per case child undertook CANTAB testing; wherever possible, community controls were prioritized over sibling controls in order to maximize age matching.

For the MRI scans, only SAM survivors were scanned and results from the recent Brain Imaging in Normal Kids (BRINK) study in Blantyre, Malawi, were used as a reference group in place of controls^(^^27^^)^.

### Variables

#### Cognitive function

Cognitive function was assessed by reported school achievement and using the CANTAB^(^^17^^)^. School achievement was assessed by current/highest school grade because, in Malawi, graduation to the next grade is dependent on passing exams rather than dictated by a child’s age. CANTAB is a widely used, well-validated tool suitable for children aged 4 years or above, with various tests covering three cognitive domains: visual memory, visual attention and working memory/planning^(^^28^^)^. We used a subset of tests, selected to examine a range of cognitive functions and to allow us to compare our results with a previous CANTAB study in Malawi^(^^18^^)^. Test do not require the ability to read any numerical or alphabetical values and are described in Table 1.

**Table 1:** Description of tests in the CANTAB assessment, presented in the order of administration used in the present study

Test	Cognitive domain	Description
1. Motor Screening Test (MOT)	Working memory/planning	Largely used to familiarize the child with the computer touch screen, the child must touch a series of crosses (×) when they appear on the screen
2. Paired Associates Learning (PAL)	Visual memory	Boxes are displayed on the screen with different shapes inside them. Each shape is displayed randomly for a number of seconds and then removed. The child needs to remember which shape is in each box. More boxes are added to increase test complexity as the child progresses
3. Pattern Recognition Memory (PRM)	Visual Memory	Random characters are displayed on the screen one after the other. At the end of the sequence, each of the characters is then displayed beside another character that was not displayed. The child needs to remember which was displayed
4. Big/Little Circle (BLC)	Visual attention	Two circles are displayed on the screen, one big and the other little. The child needs to touch on one of the circles, this is followed by a confirmation of whether it is correct or not. When the ‘rule’ changes and the other circle is correct, the child must learn and then adapt
5. Intra/Extradimensional Set Shift (IED)	Visual attention	A continuation of BLC. Two objects are displayed on the screen inside boxes. First, the child has to guess which object is correct. If s/he gets it correct, s/he has to press that object continuously. When the ‘rule’ changes, s/he will get a message that the object is incorrect and must adapt

CANTAB, Cambridge Neuropsychological Testing Automated Battery.

#### Brain structure (MRI)

Brain structure was assessed by a brain MRI scan on a subset of participants. The subset of SAM survivors selected for MRI was dictated by the availability of the MRI machine and the child who had a study appointment on that day. If there was more than one child appointment on that day, priority was given to the older child as s/he was more likely to remain still for the duration of the scan (no sedation was used). The scan was conducted following the BRINK study protocol, using a 0·35T GE Signa Ovation scanner (Sag T1 FLAIR, Ax T2 FRSE, Cor T2 FRSE, Ax DWI scans)^(^^27^^)^. Scans were reviewed by a consultant radiologist using the BRINK standard list of possible abnormalities. When abnormalities were noted, children were referred to a child neurologist for further assessment and treated as needed.

### Sample size

Sample size was predetermined by the cohort size and survival. Community controls were more difficult to recruit than were cases because they had no previous personal connection with the study team and were restricted to the number of eligible children in the family and community. The achieved sample size was expected to be powered at 90 % to detect a *Z*-score difference of 0·5 between the cases and controls for height-for-age, the main study outcome^(^^8^^)^. The sample size required to detect differences in CANTAB outcomes was not known due to lack of previous data. However, the sample size achieved was similar to that of a previous nutrition study with CANTAB outcomes in Malawi, where 418 children were recruited at baseline and test outcomes were compared for 100 *v*. ninety children at 1-year follow-up^(^^18^^)^. We aimed for a much larger sub-sample size for MRI scans than has previously been done in SAM studies, however financial constraints were also a key consideration^(^^21^^)^. Our sample size was half the size of the number scanned in the BRINK trial, which was studying the general population^(^^27^^)^.

### Analysis

Multivariable ordered logistic regression was used to assess differences in school grade between SAM survivors and controls, adjusted for *a priori* potential confounders (age, sex, HIV status and socio-economic status (SES)). An analysis additionally adjusting for height-for-age *Z*-score (HAZ) is also presented, given the known association between stunting and school achievement. Simple and multivariable linear regression was used to assess differences in CANTAB test scores between SAM survivors and controls; as well as the association with HAZ, wealth quintile and severity of SAM at admission. The test ‘IED total stages completed’ was analysed using ordered logistic regression as this is an ordered, categorical, outcome variable. Differences in the prevalence of apparent brain abnormalities between SAM survivors and the Malawian reference population were assessed using simple logistic regression. Associations between brain abnormalities and potential confounders within the group of SAM survivors were also assessed in the same manner. All analyses were conducted with the statistical software package Stata release 14.

## Results

### Cognitive function

Using ordered logistic regression, we found SAM survivors were significantly more likely to be in a lower school grade than age-matched community controls, either with or without adjustment for age, sex, HIV status, HAZ and SES (Table 2).

**Table 2:** Results of ordered logistic regression analysis comparing school grade achieved for SAM survivors *v*. controls (reference), seven years after surviving an episode of SAM, in a cohort of Malawian children

	SAM survivors (*n* 315)		Community controls (*n* 178)		Unadjusted OR	95 % CI	*P*	Adjusted OR†	95 % CI	*P*	Adjusted OR‡ incl. stunting	95 % CI	*P*
	Mean	sd	Mean	sd									
School grade achieved	2·5	1·3	3·1	1·6	0·50	0·40, 0·70	<0·0001	0·4	0·3, 0·6	<0·0001	0·54	0·35, 0·81	0·003

SAM, severe acute malnutrition.

† Adjusted for age, sex, HIV status and socio-economic status.

‡ Adjusted for height-for-age *Z*-score, age, sex, HIV status and socio-economic status. Community controls are age- and sex-matched.

**Fig. 1:**
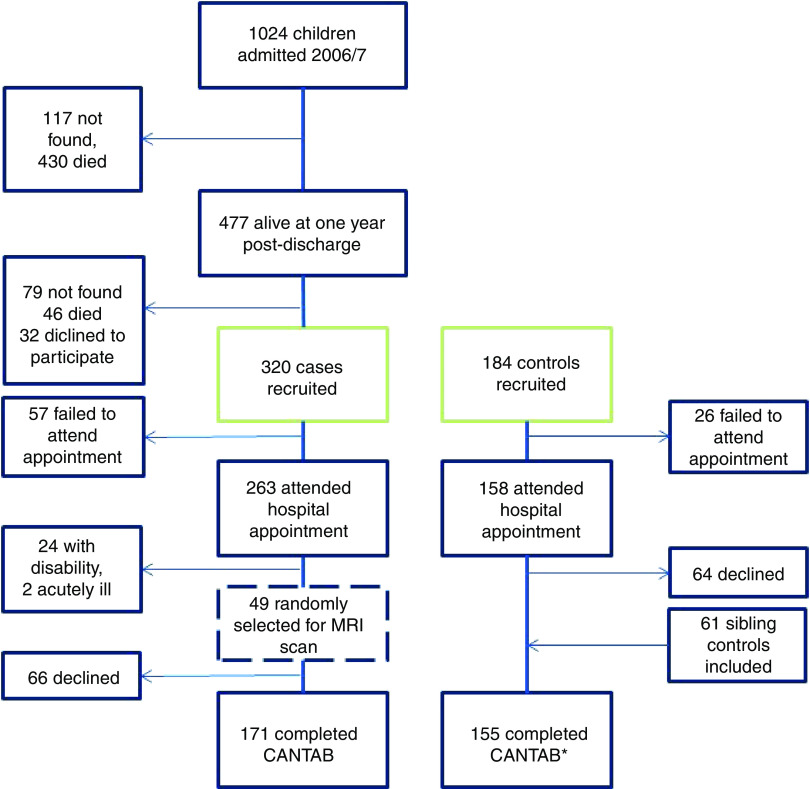
(colour online) Recruitment flow diagram for brain structure and cognitive function outcomes among Malawian children. *Of the 155 CANTAB controls, ninety-four were community children and sixty-one were siblings (CANTAB, Cambridge Neuropsychological Testing Automated Battery)

For CANTAB cognitive testing, 171 SAM survivors and 155 controls completed the tests (ninety-four were community controls and sixty-one were sibling controls; Fig. 1). Inter-group comparison showed SAM survivors had on average worse scores in all eight test outcomes (Table 3). This was statistically significant for BLC (visual attention), PRM (visual memory) and IED (visual attention) when adjusted for age only. However, only BLC was statistically significantly different after adjusting for sex, HIV and SES. No outcomes were statistically significantly different after additional adjusting for HAZ. Note the large standard deviations for most outcomes suggest that a greater sample size would be necessary to detect any potential (smaller) difference. For results disaggregated by sibling and community controls, see the online supplementary material, Supplemental Table 1.

**Table 3:** Results of regression analyses comparing outcomes of CANTAB tests for SAM survivors *v*. controls, seven years after surviving an episode of SAM, in a cohort of Malawian children

CANTAB outcome†	SAM survivors (*n* 171)		Controls (*n* 155)		SAM survivors *v*. controls, adjusted only for age			SAM survivors *v*. controls, adjusted for age, sex, HIV, SES			SAM survivors *v*. controls, adjusted for HAZ, age, sex, HIV, SES		
	Mean	sd	Mean	sd	Difference	95 % CI	*P*	Difference	95 % CI	*P*	Difference	95 % CI	*P*
BLC % correct	94·0	13·1	97·8	5·4	−4·02	−6·5, −1·6	0·001^*^	−3·35	−6·1, −0·6	0·02^*^	−1·78	−4·1, 0·5	0·13
IED total errors (adjusted)	93·7	81·5	75·3	77·3	18·34	−1·1, 37·7	0·06	6·79	−13·5, 27·1	0·51	3·75	−16·8, 24·3	0·72
MOT mean error	10·0	2·8	9·8	2·9	0·15	−0·5, 0·8	0·64	0·31	−0·4, 1·0	0·37	0·29	−0·4, 1·0	0·41
MOT mean latency (ms)	1347	502	1257	423	86·4	−16·0, 188·8	0·09	52·9	−60·0, 166·0	0·36	22·3	−90·2, 134·8	0·70
PAL total errors (adjusted)	111·1	70·4	96·3	71·7	13·9	−1·5, 29·4	0·08	8·04	−8·8, 24·9	0·35	5·57	−11·4, 22·5	0·52
PAL total errors (six shapes, adjusted)	31·2	19·7	28·4	20·8	2·61	−1·8, 7·1	0·25	1·23	−3·6, 6·1	0·62	0·59	−4·3, 5·5	0·81
PRM % correct	63·6	16·0	69·5	16·3	−4·36	−8·0, −0·71	0·02^*^	−3·73	−7·6, 0·1	0·06	−3·6	−7·5, 0·3	0·07
IED total stages completed (ordered logistic)	5·77	3·5	6·5	3·3	−0·46	−0·9, −0·02	0·04^*^	−0·32	−0·8, 0·2	0·21	−0·24	−0·8, 0·3	0·35

CANTAB, Cambridge Neuropsychological Testing Automated Battery; SAM, severe acute malnutrition; SES, socio-economic status; HAZ, height-for-age *Z*-score; BLC, Big/Little Circle; MOT, Motor Screening Test; PAL, Paired Associated Learning; PRM, Pattern Recognition Memory; IED, Intra/Extradimensional Set Shift.

* Indicates significant difference (*P* < 0·05). Test outcomes quantifying the number of total errors are adjusted for incomplete tests, as participants who fail at earlier stages of the test have fewer opportunities to make errors.

† Linear regression used unless otherwise stated.

As stunting is known to be associated with poorer cognitive outcomes, we also assessed the performance of the CANTAB tool by performing the regression of *v*. HAZ (Table 4). Results show that for every unit increase in HAZ, mean latency for the MOT test was 66 ms quicker (*P* = 0·009), after adjusting for age, sex, HIV status and SES. Other test outcomes were not significantly associated with HAZ after adjustment. Associations between test results and wealth quintiles can be found in the online supplementary material, Supplemental Table 2. There were no significant associations of severity of oedema at admission (grade 1–3), nor mid-upper arm circumference at admission in non-oedematous cases, with CANTAB outcomes (Supplemental Table 3), suggesting no evidence of a ‘dose effect’ of SAM severity at admission. However, sample size for this sub-analysis is small.

**Table 4:** Association between CANTAB cognitive testing outcomes and HAZ for the whole cohort of Malawian children (*n* 326)

CANTAB outcome	Unadjusted regression of CANTAB outcomes *v*. HAZ			Adjusted (age, sex, HIV, SES) regression of CANTAB outcomes *v*. HAZ		
	Unit difference	95 % CI	*P*	Unit difference	95 % CI	*P*
BLC % correct	0·80	−0·18, 1·79	0·11	0·94	−0·10, 1·97	0·08
IED total errors	−0·61	−10·0, 8·80	0·90	2·05	−7·25, 11·35	0·66
MOT mean error	−0·24	−0·52, 0·05	0·11	−0·24	−0·50, 0·07	0·13
MOT mean latency (ms)	−60·5	−107, −13·2	0·01^*^	−66·0	−115, −16·7	0·009^*^
PAL total errors	−0·27	−7·5, 6·9	0·94	0·53	−6·9, 8·0	0·88
PAL total errors (six shapes)	−0·21	−2·3, 1·8	0·84	0·06	−2·1, 2·2	0·95
PRM % correct	0·65	−1·15, 2·45	0·48	0·48	−1·25, 2·22	0·58

CANTAB, Cambridge Neuropsychological Testing Automated Battery; HAZ, height-for-age *Z*-score; SES, socio-economic status; BLC, Big/Little Circle; MOT, Motor Screening Test; PAL, Paired Associated Learning; PRM, Pattern Recognition Memory.

* Indicates significant difference (*P* < 0·05).

#### Brain structure (MRI)

Forty-nine survivors of SAM underwent brain MRI scans (Fig. 1). Of them, 51 % (25/49) had an MRI scan with an abnormality, the majority of which was sinusitis (43 %; 21/49). Other abnormalities detected included gliosis (8 %; 4/49) and chronic stroke (2 %; 1/49; see full descriptions in Table 5). Results were similar to those found in the BRINK study reference population where 46 % (44/96) of children had an MRI scan with an abnormality; similarly, the majority of the abnormalities were sinusitis (29 %; 28/96); 17 % (16/96) of children had an abnormal brain structure^(^^27^^)^.

**Table 5:** Summary of MRI brain scan abnormalities detected in SAM survivors in the cohort of Malawian children

MRI finding	*n*	Sex	Age (years)	HIV status
Pan sinusitis	10	5 F	Mean: 9·9	8 negative
		5 M	Range: 8–15	2 positive
Spheno-ethmoidal sinusitis	3	1 F	Mean: 10·0	1 negative
		2 M	Range: 8–13	2 positive
Sphenoid and maxillary sinusitis	2	F	Mean: 8·5	negative
			Range: 8–9	
Ethmoid and maxillary sinusitis	3	2 F	Mean: 9·6	1 negative
		1 M	Range: 8–12	2 positive
Frontal sinusitis	1	M	8	negative
Maxillary sinusitis	1	M	7	negative
Gliosis in subcortical white matter of frontal lobes	1	M	7	negative
Gliosis of cerebellum and pan sinusitis	1	F	10	positive
Gliosis of cerebellum	1	F	10	negative
Peritrigonal gliosis	1	F	8	negative
Chronic stroke of the left putamen and caudate head	1	F	11	positive
Summary – all abnormalities	25	13 F	Mean: 9·1	17 negative
		11 M	Range: 7–15	8 positive

SAM, severe acute malnutrition; F, female; M, male.

The OR of having any brain abnormality including sinusitis was 1·23 (95 % CI 0·62, 2·44) for survivors of SAM compared with the BRINK controls (*P* = 0·55); when eliminating sinusitis, the OR for abnormal brain structure for SAM survivors compared with BRINK controls was 0·57 (95 % CI 0·20, 1·60; *P* = 0·30). There was also no significant association between brain abnormalities and other potential risk factors, including HIV status, age, sex and SES.

## Discussion

SAM survivors were more likely to be in a lower grade at school than community control children. SAM was also associated with consistently poorer scores in all CANTAB cognitive tests, significantly so in areas of visual attention and visual memory, although adjusting for HIV and SES diminished most of the statistically significant differences. SAM survivors did not have increased odds of gross structural brain abnormalities compared with ‘normal’ Malawian children.

Our observation of long-term cognitive deficits in SAM survivors adds to other abnormalities including stunting, underweight, lack of lean mass and diminished muscle strength previously reported in this group^(^^8^^)^. The absence of long-term structural brain changes (despite functional impairments) compared with the national comparison group also concurs with results from previous studies which found that abnormalities observed during and shortly after SAM did not persist after treatment^(^^20^^,^^21^^)^. Head circumferences of SAM survivors in this cohort also did not differ significantly compared with community controls seven years post-SAM (51·1 *v*. 52·1 cm respectively; *P* = 0·12)^(^^8^^)^. Although brain structure of SAM survivors did not appear to differ from normal Malawian children, it is important to note the high rate of incidental MRI abnormalities in both SAM survivors and the reference group when compared with US populations^(^^27^^)^. Sinusitis was especially prevalent and although this is not uncommon among radiology scans in children^(^^29^^)^, its presence may highlight a high background burden of infection and chronic inflammation and has been found to be associated with exposure to indoor cooking smoke^(^^27^^,^^30^^)^.

For CANTAB cognitive testing, SAM survivors scored significantly worse in areas of visual memory and visual attention. SAM survivors had worse mean scores in all tests; this could be an indication that they also perform worse on average in school exams, which may explain the significantly lower school grade achieved. There is also evidence that children who are stunted can be entered into school later, kept in lower grades at school or socially interacted with in less advanced ways, because they appear or behave younger than their contemporaries, which would also affect SAM survivors^(^^31^^,^^32^^)^.

After adjusting for HIV and SES, differences in CANTAB scores were not statistically significant, except for the BLC visual attention test. This suggests that much of the cognitive impairment in the SAM survivors is due to the confounding effects of HIV and SES, or that these variables lie on the casual pathway. In practical terms, whether directly or indirectly related to the nutritional insult, an episode of SAM still indicates a child who is at risk of poor cognitive function. The implications of HIV are another clear indication of the ‘vicious cycle’ of infection and malnutrition^(^^33^^)^. The effect of survivor bias in the SAM survivors and the evolutionary adaptation of ‘brain sparing’ development may also explain the diminished effect size, after adjustment, between cases and controls^(^^34^^)^. It is interesting that HAZ is not statistically associated with CANTAB scores. However, this may be explained by the high prevalence of stunting across the whole sample, including controls (mean HAZ = −1·6 (sd 1·2)).

A false negative in the adjusted CANTAB results should also be considered due to potentially suboptimal sample size. For the great majority of our cohort, this was the first time they had used a computer and therefore the learning curve associated with this likely added ‘noise’ to the resulting data. This may explain the large standard deviations present across the test scores, necessitating a larger sample size. For example, for PAL total errors, a *post hoc* sample size calculation suggests that, with 5 % significance and 80 % power, a sample size of 362 in each group would be needed to demonstrate a statistically significant difference^(^^35^^)^.

To understand how our population performed against children in other contexts, we compared CANTAB results in our study with others from the literature (Table 6)^(^^18^^,^^28^^)^. Nkhoma *et al*. used CANTAB to test cognitive improvements following a school feeding programme in Malawi^(^^18^^)^. One of the thirteen CANTAB outcomes presented was significantly improved between the feeding group and controls (IED Pre-ED errors reduced, *P* = 0·02). If we compare our SAM survivors and controls between the ages of 6 and 8 years with Nkhoma’s control group results at baseline, we see some relatively large differences in scores, although with no clear trend as to which children did better or worse – perhaps, again, results of large standard deviations. When compared with published data from UK schoolchildren of the same age^(^^28^^)^, we find that UK scores are generally better than those in Malawi. With UK children very used to computers relative to this cohort of Malawi children, this is not unexpected. The outlier (MOT) could be due to chance or due to differences in coaching since it is the first test in the battery.

**Table 6:** Comparison of mean scores on CANTAB tests for children in other studies in Malawi and the UK with those of SAM survivors in the present study (aged 6–8 years only)

CANTAB outcome	Malawian children aged 6–8 years^(^^18^^)^ (*n* 111)		UK schoolchildren aged 6–8 years^(^^28^^)^ (*n* 198)		Malawian children aged 6–8 years, present study			
					SAM survivors (*n* 35)		Controls (*n* 29)	
	Mean	sd	Mean	sd	Mean	sd	Mean	sd
MOT mean errors	N/A		22·0	17·0	9·45	2·4	11·1	2·8
MOT mean latency	N/A		873·7	188·0	1431·5	605·9	1242·5	465·3
PAL total errors (adjusted)	74·8	17·9	N/A		117·1	77·8	99·0	82·8
PRM % correct	N/A		83	10·7	59·2	14·7	68·6	17·2
IED total errors (adjusted)	157	69·9	N/A		99·6	82·5	67·5	58·8
IED stage completed	3·4	3·2	7·66	2·1	5·6	3·7	6·8	3·0

CANTAB, Cambridge Neuropsychological Testing Automated Battery; SAM, severe acute malnutrition; MOT, Motor Screening Test; PAL, Paired Associated Learning; PRM, Pattern Recognition Memory; IED, Intra/Extradimensional Set Shift; N/A, results were not presented for these test outcomes.

We acknowledge some limitations of our study. First, ‘healthy survivor’ bias is vital to note and will likely have affected all our study outcomes, as many of our original cohort of SAM children died soon after admission or in the year after. Only 352/1024 of those originally admitted were still alive for follow-up at this 7-year stage. Those whose brain structure and cognitive function were most affected by SAM are also those who have likely died and thus our results are likely to be an underestimate of the adverse impact of SAM on cognition and related outcomes at the population level.

Second, we do not have data on prenatal nutritional status or birth weight. These could be potential confounders and SAM may be a symptom of other underlying problems rather than directly causing impairments itself.

For MRI scans, selection bias may have played a part since children or carers who were nervous of the scan process were less likely to consent to a scan. We also acknowledge that subtle but clinically important changes in brain volume would not necessarily have been identified in our study; future work may quantify brain volume to explore this issue in more detail.

Finally, this SAM population differs from cohorts treated today and hence results are not directly generalizable. Whereas all our children were initially treated as inpatients, today’s programmes focus on early identification and treatment through community management of acute malnutrition. They also use WHO growth standards for admission whereas, in 2006, we used the National Center for Health Statistics growth references, per national protocols^(^^36^^)^.

Balancing these limitations, our study also has many strengths. It is one of very few that has looked in detail at such a wide range of outcomes following SAM. It is also rare to get a follow-up period of this length post-SAM. Most importantly, we have generated baseline data for relatively novel assessment tools, such as CANTAB, which can be used to inform the design of future studies. Ideally, these will be intervention studies that seek to support children affected by SAM to not only survive but thrive.

## Conclusion

In conclusion, based on school achievement and trends in CANTAB cognitive test results, SAM survivors likely have impaired cognitive function, especially in visual memory and visual attention, compared with controls, seven years post-discharge from treatment. However, there was no evidence of gross alterations in brain structure using MRI scans. Whether the cause of impaired school achievement and cognitive function is biological or social warrants further exploration given the apparent preservation of brain structure, although this should not detract from the practical importance of poorer school achievement. The use of CANTAB as a novel cognitive testing tool was popular and feasible in this field setting; however, these results suggest that sample size in future studies, especially in computer-naïve contexts, may need to be larger (>300 per group) to detect significant inter-group differences.

## Supplementary material

For supplementary material accompanying this paper visit https://doi.org/10.1017/S1368980018003282. click here to view supplementary material
