# Supplementary material for: Brain MRI and cognitive function seven years after surviving an episode of severe acute malnutrition in a cohort of Malawian children
Source: Public Health Nutr. 2018 Dec 3;22(8):1406–14. Online ahead of print. doi: 10.1017/S1368980018003282 (PMC6411134; doi:10.1017/S1368980018003282)
Supplement: Supplementary file 1 [file S1368980018003282sup001.docx]

# Supplementary Material

**Supplemental Table 1**: Result of regression analysis for controls compared to SAM survivors for outcomes of the “CANTAB” cognitive function tests, disaggregated by sibling and community controls

| **CANTAB** | **Cases** | **Sibling** | | | **Community** | | |
| --- | --- | --- | --- | --- | --- | --- | --- |
|  | **Mean** (SD)  N=171 | **Mean** (SD)  N=61 | **Difference adjusted for age only**  (95% CI)  *P value* | **Difference adjusted⸸**  (95% CI)  *P value* | **Mean** (SD)  N=94 | **Difference adjusted for age only**  (95% CI)  *P value* | **Difference adjusted⸸**  (95% CI)  *P value* |
| BLC Percent Correct | 94.0 (13.1) | 98.4 (4.1) | 5.65  *(1.9, 9.4)*  *0.004 ** | 5.16  *(1.2, 9.1)*  *0.01 ** | 97.5 (5.9) | 3.37  *(0.7, 6.1)*  *0.02 ** | 2.51  *(-0.5, 5.6)*  *0.11* |
| IED Total Errors (adjusted) | 93.7 (81.5) | 92.2 (84.3) | 3.18  *(-26, 33)*  *0.83* | 4.77  *(-24.5, 34)*  *0.75* | 67.8 (73.2) | -26.8  *(-48, -5.9)*  *0.01** | -12.04  *(-34, 10.4)*  *0.29* |
| IED Stage completed (ordinal logistic regression‡) | 5.77 (3.5) | 5.85 (3.7) | -0.04  *(-0.7, 0.7)*  *0.90* | -0.03  *(-0.8, 0.7)*  *0.93* | 6.81  (3.1) | 0.66  *(0.2, 1.2)*  *0.009** | 0.47  *(-0.1, 1.0)*  *0.098* |
| MOT mean latency | 1348 (502) | 1282 (411) | -39.6  *(-178, 100)*  *0.58* | -18.5  *(-163, 126)*  *0.80* | 1240  (431) | -115.4  *(-233, 2.90)*  *0.06* | -78.7  *(-210, 53)*  *0.24* |
| MOT mean error | 10.0 (2.8) | 10.2 (3.2) | 0.28  *(-0.6, 1.1)*  *0.52* | 0.21  *(-0.6, 1.1)*  *0.63* | 9.6  (2.7) | -0.41  *(-1.1, 0.3)*  *0.26* | -0.71  *(-1.5, 0.1)*  *0.08* |
| PAL total errors (adjusted) | 111.1 (70) | 116.4 (77) | 10.5  *(-10, 31)*  *0.32* | *12.1*  *(-9.3, 33)*  *0.27* | 83.4 (65) | -28.9  *(-46, -11)*  *0.001** | *-23.0*  *(-42, -3.6)*  *0.02 ** |
| PAL total errors (6 shapes adjusted) | 31.2  (20) | 32.9 (20) | 2.82  *(-3.2, 8.9)*  *0.36* | 3.02  *(-3.2, 9.2)*  *0.34* | 25.5 (21) | -5.9  *(-11, -0.9)*  *0.02** | -4.38  *(-10.0,1.2)*  *0.12* |
| PRM percent correct | 63.6 (16) | 73.2 (17) | 5.69  *(0.4, 10.9)*  *<0.04** | 4.42  *(-0.8, 9.7)*  *0.097* | 67.4 (15) | *3.73*  *(-0.3, 7.8)*  *0.07* | 3.34  *(-1.0, 7.7)*  *0.13* |

Linear regress unless otherwise stated‡; adjusted differences⸸ includes age, sex, HIV status and socioeconomic status in the model. Test outcomes which quantify the number of total errors have been adjusted for incomplete tests as subjects who fail at earlier stages of the test have fewer opportunities to make errors.

**Supplemental Table 2**: Associations between CANTAB cognitive test results and wealth quintile

| **CANTAB outcome** | **Wealth quintile (1=poorest)** | **Mean** | **SD** | **Unadjusted Coefficient** | **P value** |
| --- | --- | --- | --- | --- | --- |
| BLC % correct | 1 | 95.8 | 8.1 | *Ref* | *Ref* |
|  | 2 | 96.7 | 5.1 | 0.91 (-2.9 to 4.8) | 0.65 |
|  | 3 | 95.9 | 10.0 | 0.07 (-4.0 to 4.2) | 0.97 |
|  | 4 | 96.6 | 9.4 | 0.81 (-2.9 to 4.5) | 0.67 |
|  | 5 | 94.2 | 16.6 | -1.57 (-5.5 to 2.4) | 0.43 |
| IED total errors (adjusted for incomplete tests⸸) | 1 | 118.3 | 85.8 | *Ref* | *Ref* |
|  | 2 | 84.1 | 77.7 | -34.2 (-63.1 to -5.3) | 0.02 |
|  | 3 | 69.8 | 71.0 | -48.9 (-78.9 to -18.0) | 0.002 |
|  | 4 | 90.3 | 80.9 | -27.9 (-55.6 to -0.3) | 0.05 * |
|  | 5 | 50.4 | 62.1 | -67.9 (-97.4 to -38.4) | <0.001* |
| MOT mean error | 1 | 10.2 | 3.1 | *Ref* | *Ref* |
|  | 2 | 10.2 | 2.9 | 0.05 (-0.90 to 0.99) | 0.92 |
|  | 3 | 9.5 | 2.7 | -0.69 (-1.68 to 0.31) | 0.18 |
|  | 4 | 9.5 | 2.6 | -0.69 (-1.61 to 0.23) | 0.14 |
|  | 5 | 10.1 | 2.8 | -0.02 (-1.00 to 0.96) | 0.96 |
| MOT mean latency (millisecs) | 1 | 1381 | 474 | *Ref* | *Ref* |
|  | 2 | 1329 | 492 | -52.0 (-209 to 105) | 0.52 |
|  | 3 | 1203 | 379 | -179 (-344 to -13.3) | 0.03 * |
|  | 4 | 1306 | 485 | -75.4 (-228 to 77.2) | 0.33 |
|  | 5 | 1283 | 497 | -98.6 (-261 to 64.2) | 0.23 |
| PAL total errors (adjusted for incomplete tests⸸) | 1 | 120.2 | 67.6 | *Ref* | *Ref* |
|  | 2 | 108.9 | 71.3 | -11.3 (34.9 to 12.3) | 0.35 |
|  | 3 | 95.8 | 60.9 | -24.5 (-49.4 to 0.53) | 0.06 |
|  | 4 | 100.3 | 78.0 | -19.9 (-43.0 to 3.13) | 0.09 |
|  | 5 | 86.6 | 73.1 | -33.6 (-58.2 to -9.03) | 0.008 * |
| PAL total errors (6 shapes, adjusted) | 1 | 33.4 | 19.8 | *Ref* | *Ref* |
|  | 2 | 31.1 | 20.2 | -2.31 (-9.0 to 4.4) | 0.50 |
|  | 3 | 30.5 | 21.0 | -2.89 (-10.0 to 4.2) | 0.42 |
|  | 4 | 28.4 | 20.1 | -4.98 (-11.6 to 1.60) | 0.14 |
|  | 5 | 23.7 | 20.2 | -9.70 (-16.7 to -2.6) | 0.007 * |
| PRM % correct | 1 | 60.1 | 14.7 | *Ref* | *Ref* |
|  | 2 | 63.7 | 15.8 | 3.69 (-2.1 to 9.4) | 0.21 |
|  | 3 | 67.8 | 16.7 | 7.72 (1.7 to 13.7) | 0.01 |
|  | 4 | 71.6 | 17.1 | 11.5 (6.0 to 17.1) | <0.001* |
|  | 5 | 69.1 | 15.1 | 9.07 (3.1 to 15.0) | 0.003 * |
| IED total stages completed (ordered logistic‡) | 1 | 4.69 | 3.7 | *Ref* | *Ref* |
|  | 2 | 6.13 | 3.4 | 0.71 (0.0 to 1.4) | 0.04 * |
|  | 3 | 6.79 | 3.1 | 1.21 (0.5 to 1.9) | 0.001 * |
|  | 4 | 5.90 | 3.4 | 0.65 (0.0 to 1.3) | 0.05 * |
|  | 5 | 7.63 | 2.7 | 1.98 (1.2 to 2.7) | <0.001* |

Linear regress unless otherwise stated‡. Test outcomes which quantify the number of total errors have been adjusted for incomplete tests as subjects who fail at earlier stages of the test have fewer opportunities to make errors⸸

**Supplementary Table 3**: Association between CANTAB cognitive testing outcomes and severity of SAM at admission (oedema classification and mid-upper arm circumference)

| CANTAB outcomes | Adjusted (age, sex, HIV, SES) regression of CANTAB outcomes on oedema severity (1, 2 or 3) n=110 | | Adjusted (age, sex, HIV, SES) regression of CANTAB outcomes on MUAC at admission (if no oedema) n=72 | |
| --- | --- | --- | --- | --- |
|  | Unit Difference (CI) | P value | Unit Difference (CI) | P value |
| BLC % correct | 2.57 (-1.2, 6.4) | 0.19 | 0.66 (-3.7, 5.0) | 0.75 |
| IED total errors | 6.14 (-17.2, 29.5) | 0.60 | -18.31 (-48.3, 11.6) | 0.22 |
| MOT mean error | 0.04 (-0.7, 0.8) | 0.91 | -0.36 (-1.3, 0.6) | 0.44 |
| MOT mean latency (millisecs) | -56.9 (-176.5, 62.6) | 0.35 | -159.2 (-356.7, 38.4) | 0.11 |
| PAL total errors | -14.85 (-33.0, 3.3) | 0.11 | -0.47 (-24.2, 23.3) | 0.97 |
| PAL total errors (6 shapes) | -3.29 (-8.4, 1.8) | 0.21 | -0.64 (-7.7, 6.4) | 0.85 |
| PRM % correct | -2.62 (-6.7, 1.6) | 0.22 | 2.45 (-4.2, 9.1) | 0.46 |

Linear regression of CANTAB outcomes against oedema severity score (1 to 3), and for those without oedema, mid-upper arm circumference at admission. * indicated significant difference (p<0·05). Unadjusted analyses also showed no significant differences.
